# Directed evolution of a beta‐lactamase samples a wide variety of conformational states

**DOI:** 10.1002/pro.70322

**Published:** 2025-10-11

**Authors:** Jing Sun, Monika Timmer, Steffen Brünle, Aimee L. Boyle, Marcellus Ubbink

**Affiliations:** ^1^ Macromolecular Biochemistry, Leiden Institute of Chemistry Leiden University Leiden The Netherlands; ^2^ Biophysical Structural Chemistry, Leiden Institute of Chemistry Leiden University Leiden The Netherlands; ^3^ School of Chemistry University of Bristol Bristol UK

**Keywords:** beta‐lactamase, NMR, protein dynamics, protein evolution, x‐ray crystallography

## Abstract

In directed evolution, enzyme activity is improved in successive generations of laboratory evolution, which can be described by a simple stepwise climb toward a peak in the fitness landscape. In a naive model of evolution, it can be assumed that each enzyme variant along this path is in a single, well‐defined state that differs slightly from the previous one. We analyzed the structural changes in mutants of the β‐lactamase BlaC from *Mycobacterium tuberculosis* obtained via directed evolution for increased ceftazidime hydrolysis activity. Crystal structures of three successive mutants only show an increase in the dynamics of a loop that lines the active site (Ω‐loop), enabling better access of the large substrate. However, NMR spectra of wild type and nine mutants of different branches of the directed evolution experiment show a much more diverse and complex picture of the conformational effects. Many mutants show micro‐millisecond dynamics for certain regions and most show peak doubling, indicative of two or more conformations being populated. Thus, the straightforward climb to increased ceftazidime activity in the fitness landscape masks a complex trajectory in the conformational landscape, emphasizing the complex and epistatic interplay that single mutations can have on the structure and dynamics of enzymes.

## 1 INTRODUCTION

Directed evolution allows for rapid enhancement of enzyme activity on poor substrates (Campbell et al., 2018; Martínez & Schwaneberg, 2013; Nirantar, 2021; Vidal et al., 2023). Selection for enhanced activity can result in a stepwise, progressive climb toward a fitness peak in a limited number of generations. The underlying structural changes accompanying the change in activity can in some cases be described by a change in the population of two states, one inactive and the other active (Otten et al., 2020; St‐Jacques et al., 2023). Such a binary system may, however, not be a general situation because the effect of mutations in proteins depends on the background of all residues (epistasis) and can have unpredictable effects. Generally, protein folds are resistant to the effects of mutations and overall, the structures of the enzymes of successive generations of laboratory evolution may change little. However, subtle changes in atomic positions can already affect the atomic interactions and hydrogen bond (H‐bond) network and, thus, the activity of an enzyme (Campbell et al., 2016; Campitelli et al., 2020). Also, changes in the dynamics can strongly influence catalytic properties (Bhabha et al., 2013; Fisette et al., 2012; Fraser et al., 2009; Gobeil et al., 2014; Jackson et al., 2009; Maria‐Solano et al., 2018; Rossi et al., 2022). Though progress has been made using AI (Casadevall et al., 2023; Wayment‐Steele et al., 2024), changes in enzyme conformation remain difficult to predict and may be more complex than a progressive change in phenotype suggests. We investigated the structural adaptations and conformational dynamics that underly functional changes by directed evolution of the β‐lactamase BlaC. BlaC is a class A serine β‐lactamase found in *Mycobacterium tuberculosis* (Wang et al., 2006). The enzymes in this class utilize an active site serine (Ser70) to perform a nucleophilic attack on β‐lactam rings, forming a covalent acyl‐enzyme intermediate that is subsequently hydrolyzed (Fisher & Mobashery, 2009; Strynadka et al., 1992). BlaC can efficiently hydrolyze penicillin antibiotics but has low activity against later‐generation oxyimino‐cephalosporins, such as ceftazidime, due to their bulky C7β aminothiazole‐oxyimino side chain (Sun et al., 2023; Wang et al., 2006). Recently, we reported that ceftazidime hydrolysis is enhanced by a single mutation, P167S (Ambler numbering) (Ambler et al., 1991) in BlaC, in line with what was reported for other β‐lactamases (Papp‐Wallace et al., 2017; Poirel et al., 2001; Stojanoski et al., 2015; Sun et al., 2023). The mutation changes a *cis* peptide bond to *trans*, causing the opening of the Ω‐loop (residue 164–179) and enlarging the active site. However, although enhanced, the catalytic efficiency remains low compared to that of WT BlaC for other substrates, such as ampicillin and nitrocefin (Sun et al., 2023). Laboratory evolution of other β‐lactamases was used to enhance the catalytic efficiency against ceftazidime, and the regions affected by mutations included the Ω‐loop (Alsenani et al., 2022; Bae et al., 2006; Delmas et al., 2006; Hobson et al., 2022; Nijhuis et al., 2015; Papp‐Wallace et al., 2017; Poirel et al., 2001), the gatekeeper loop (residue 103–106) (Feiler et al., 2013), and the B3 β‐strand (residue 233–246) (Brown et al., 2020; Delmas et al., 2006; Kemp et al., 2021). A recent study on β‐lactamase OXA‐48 also improved the ceftazidime resistance through rounds of directed evolution, elucidating the molecular mechanisms underlying epistasis (Fröhlich et al., 2024).

In the current study, BlaC was evolved to enhance ceftazidime activity to explore the changes in conformations and dynamics, which were analyzed with crystallography and NMR spectroscopy. Crystal structures show increasing dynamics in the Ω‐loop along the evolutionary trajectory. The overall structure of the remaining part of the protein displayed hardly any differences. The NMR analysis showed a more intricate picture and revealed a surprising complexity of conformational states, exhibiting enhancement in μs–ms dynamics in several regions, as well as the population of multiple states for global or local parts of the protein, with patterns that changed in each successive next generation. It is concluded that during the directed evolution, many different states are visited in the conformational landscape, indicating that the destabilization of the Ω‐loop enabled access to a wide range of new conformations.

## 2 RESULTS

### 2.1 BlaC can readily be evolved to high ceftazidime activity

**FIGURE 1:**
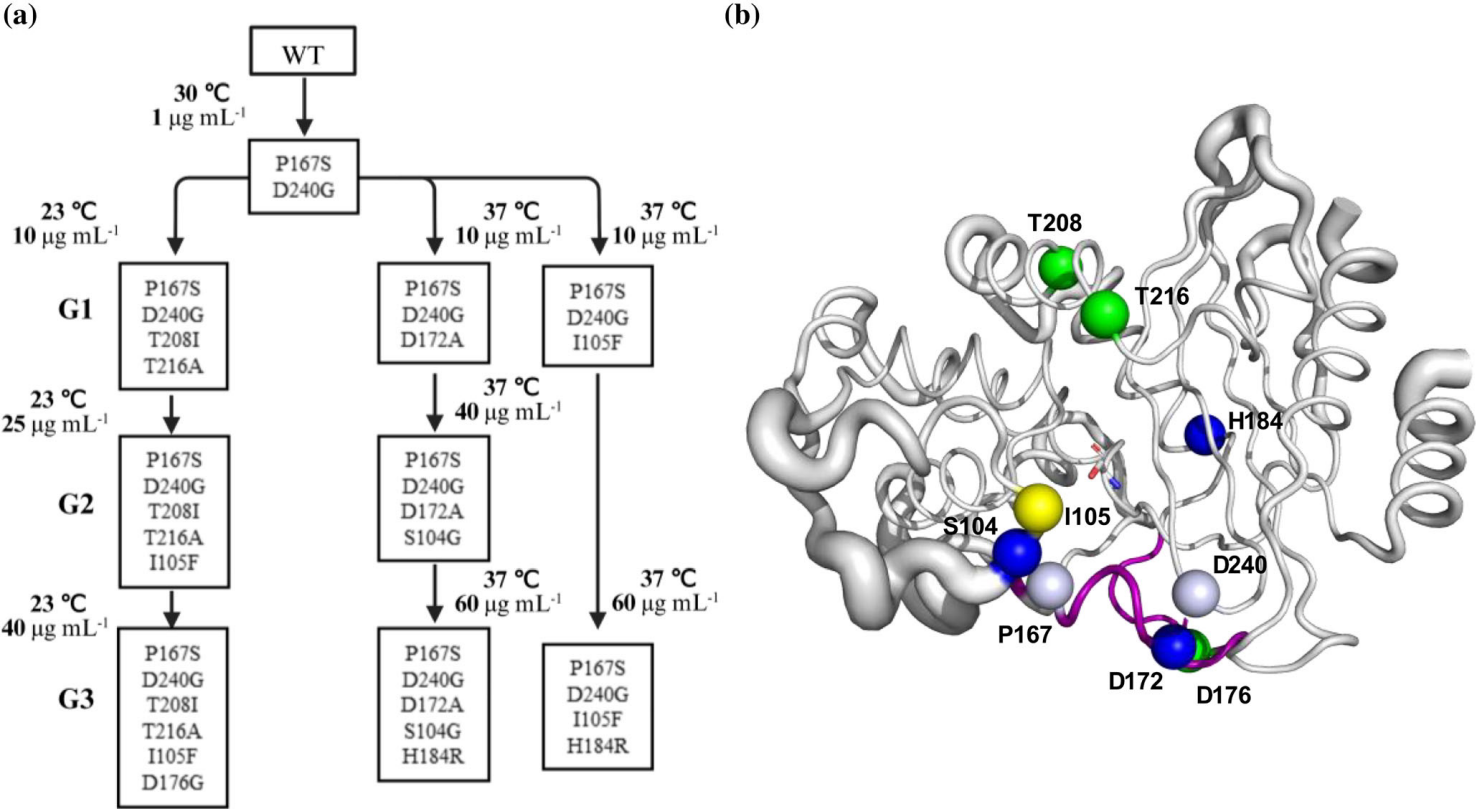
Schematic representation of the directed evolution trajectory and obtained variants. (a) Amino acid mutations obtained in successive generations are shown and the ceftazidime concentration and the temperatures used for selection are indicated. (b) The locations of the mutations (Cα atoms) acquired during selection at 23°C (T216A, T208I, D176G, green spheres, I105F, yellow sphere) and 37°C (D172A, S104G, and H184R, blue spheres, I105F, yellow) are shown on the WT BlaC structure (PDB: 2GDN; Wang et al., 2006) in tube representation and the thickness of the tubes correlates with B‐factors. The catalytic residue Ser70 is shown in sticks, the mutations in the template (P167S and D240G) are indicated as pale blue spheres, and the Ω‐loop is colored purple.

In a screen for BlaC mutants with improved ceftazidime resistance in an *Escherichia coli* expression system at three temperatures (23, 30, and 37°C), the BlaC variant P167S/D240G (PD) was found at 30°C, exhibiting a 16‐fold increased resistance for ceftazidime as compared to the wild‐type (WT). Grown at 23 and 37°C, the resistance of this PD variant was eightfold greater than that of WT (Table 1). To test how resistance would further evolve and with which trade‐offs (Noda‐Garcia & Tawfik, 2020), directed evolution was performed using the *blaC* gene of the PD variant as the template. It was decided to perform the selection at two temperatures to establish whether accessible mutational pathways would differ. A library of random mutants was generated using error‐prone PCR and three successive rounds were performed (generations G1–G3) at 23 or 37°C, using increasing ceftazidime concentrations, as indicated in Figure 1a. In every screen, 10^6^ to 10^8^ clones were sampled. In the selection performed at 23°C, G1 yielded mutant P167S/D240G/T208I/T216A (PDTT), in G2 mutation I105F was acquired (PDTTI), and in G3, D176G was added (PDTTID). At 37°C, two variants were found that were further evolved, yielding P167S/D240G/D172A/S104G/H184R (PDDSH) and P167S/D240G/I105F/H184R (PDIH) in G3, respectively (Figure 1b). The PDIH variant evolved in G2 but was sufficiently resistant to grow under the conditions of G3. After the third generation, no further improvements were obtained with this method. The minimum inhibitory concentration (MIC) was determined using drop assays of OD_600_ 0.3–0.0003 on agar plates containing a range of antibiotic concentrations. This approach is more subtle than the standard power‐of‐two MIC determination (Figures S1–S3) (van Alen et al., 2023). The mutants selected at 37°C display more than 15‐fold increased resistance compared to PD and over 120‐fold compared to WT (Table 1).

**TABLE 1:** Minimal concentrations of ceftazidime at which growth of *E. coli* producing BlaC wild‐type and mutants is not observed (MIC), determined with a droplet test. The estimated error is 5 μg/mL, based on the differences in growth between plates with increasing amounts of ceftazidime (Figures S1–S3).

Mutations	G	Selection	μg/mL		
		Temp. (°C)	23°C	30°C	37°C
WT			<0.5	<0.5	0.5
P167S/D240G (PD)	0	30	4	8	4
P167S/D240G/I105F (PDI)	1	37	8	30	16
P167S/D240G/I105F/H184R (PDIH)	3	37	15		63
P167S/D240G/T208I/T216A (PDTT)	1	23	20		55
P167S/D240G/T208I/T216A/I105F (PDTTI)	2	23	35		60
P167S/D240G/T208I/T216A/I105F/D176G (PDTTID)	3	23	63		63
P167S/D240G/D172A (PDD)	1	37	10		35
P167S/D240G/D172A/S104G (PDDS)	2	37	10		45
P167S/D240G/D172A/S104G/H184R (PDDSH)	3	37	15		63

Abbreviation: G, generation.

### 2.2 Protein stability is increased in successive generations

To characterize the BlaC variants further, the enzymes were overproduced in *E. coli* and purified. The yield of soluble protein of variant PD was reduced relative to that of WT BlaC. For the variants from the successive generations of direct evolution, the level of soluble protein increased (Figure S4a). The thermostability of all purified variants was determined in a Trp fluorescence assay. The parent variant for evolution, PD, showed a melting temperature (*T*_m_) of 52.8°C, a decrease of 9.2°C compared to WT BlaC (62°C, Figure S4b and Table 2) (van Alen et al., 2023). Each mutant selected at 37°C displayed better stability than its parent. At 23°C, a further decrease in *T*_m_ was seen in G1 (−11.3°C), followed by increasing values in the next two generations. Thus, thermostability and solubility assays show the same trend, suggesting that the stabilizing effects of the mutations contribute to the better performance of the variants in later generations (Nearmnala et al., 2021; Nirantar, 2021).

**TABLE 2:** Melting temperatures for BlaC variants.

BlaC variant	G	Selection temp. (°C)	*T*_m_ (°C)^a^	Δ*T*_m_ (SD) (mut‐WT) (°C)	Δ*T*_m_ (SD) (mut‐templ.) (°C)
WT			62.0	—	
P167S/D240G (template)	0	30	52.8	−9.2	—
P167S/D240G/T216A/T208A (PDTT)	1	23	50.7	−11.3	−2.1
P167S/D240G/T216A/T208A/I105F (PDTTI)	2	23	52.6	−9.4	−0.2
P167S/D240G/T216A/T208A/I105F/D176G (PDTTID)	3	23	53.8	−8.2	1.0
P167S/D240G/D176G (PDD)	1	37	54.0	−8.0	1.2
P167S/D240G/D176G/S104G (PDDS)	2	37	55.3	−6.7	2.5
P167S/D240G/D176G/S104G/H184R (PDDSH)	3	37	57.5	−4.5	4.7
P167S/D240G/I105F (PDI)	1	37	55.0	−7.0	2.2
P167S/D240G/I105F/H184R (PDIH)	3	37	56.8	−5.2	4.0

Abbreviation: G, generation.

^a^ The estimated error from triplet experiments in *T*_m_ is 0.1°C–0.2°C.

### 2.3 A trade‐off in ceftazidime and nitrocefin activities

**FIGURE 2:**
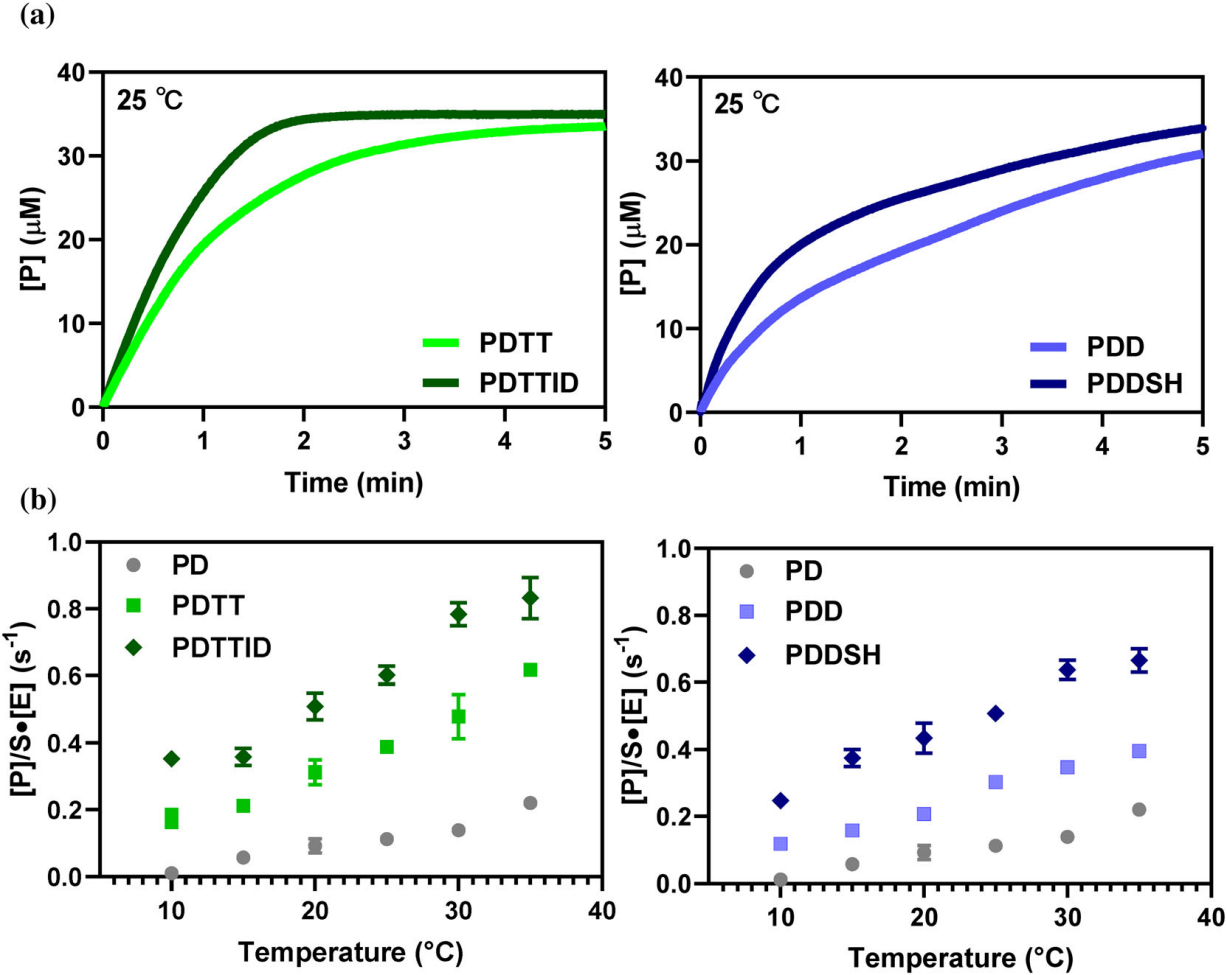
Enzyme activities. (a) Product formation curves of ceftazidime hydrolysis at 25°C for G1 and G3 variants. (b) Temperature dependence of the turnover numbers for ceftazidime hydrolysis. All experiments were performed with 1 μM enzyme in 100 mM sodium phosphate buffer, pH 6.5. Error bars represent the standard deviation of the mean of duplicate measurements.

To evaluate the β‐lactam resistance profile of the evolved BlaC mutants, kinetic parameters were determined. Increased ceftazidime activity could be accompanied by trade‐offs, therefore, both ceftazidime and nitrocefin (Structures are shown in Figure S5) hydrolysis rates were measured. It is noted that nitrocefin is a chromogenic substrate and not antibiotic used in the clinic. However, it is suitable to detect trade‐off effects. Nitrocefin hydrolysis exhibits classical (apparent) (Sun et al., 2023) Michaelis–Menten kinetics (Figure S6a). The catalytic efficiencies ($k_{\mathrm{cat}}/K_{M}^{\mathrm{app}}$) decrease up to 150‐fold for the variants, compared to WT BlaC (Table S1), indicating that the directed evolution for enhanced ceftazidime resistance compromised nitrocefin activity. The reaction kinetics for the hydrolysis of ceftazidime are more complex, as was also observed for BlaC P167S (Sun et al., 2023). In the first 5 min, the product formation curve exhibits biphasic kinetics for BlaC variants PDD and PDDSH, with an initial fast phase, followed by a slower, linear phase (Figure 2a). The BlaC variants PDTT and PDTTID variants hydrolyzed ceftazidime so rapidly that two phases could not be distinguished. Thus, at least in some of the variants, classical Michaelis–Menten kinetics are not applicable, and therefore, to compare the relative activities under these specific conditions, we defined a catalytic turnover number as the initial rate of product (*P*) formation in this assay, measured from the initial slope of the product curve, per enzyme (E) molecule, $\frac{\left[P\right]}{\text{time}\left(s\right)\cdot \left[E\right]}$ (s^−1^). The third‐generation variants showed a 4‐ to 35‐fold increase in catalytic turnover compared to the parent mutant PD, and a 55‐ to 255‐fold increase compared to the WT at 25°C (Figure 2b and Table S2). The catalytic turnover numbers increase monotonously with temperature to 35°C, equally for the mutants selected at 23 and 37°C (Figures 2b and Figure S6b), indicating that the former ones do not suffer from short‐term stability problems at this temperature, in line with the in‐cell results (Figure S1).

### 2.4 Directed evolution is accompanied by a complex variety of conformational states

**FIGURE 3:**
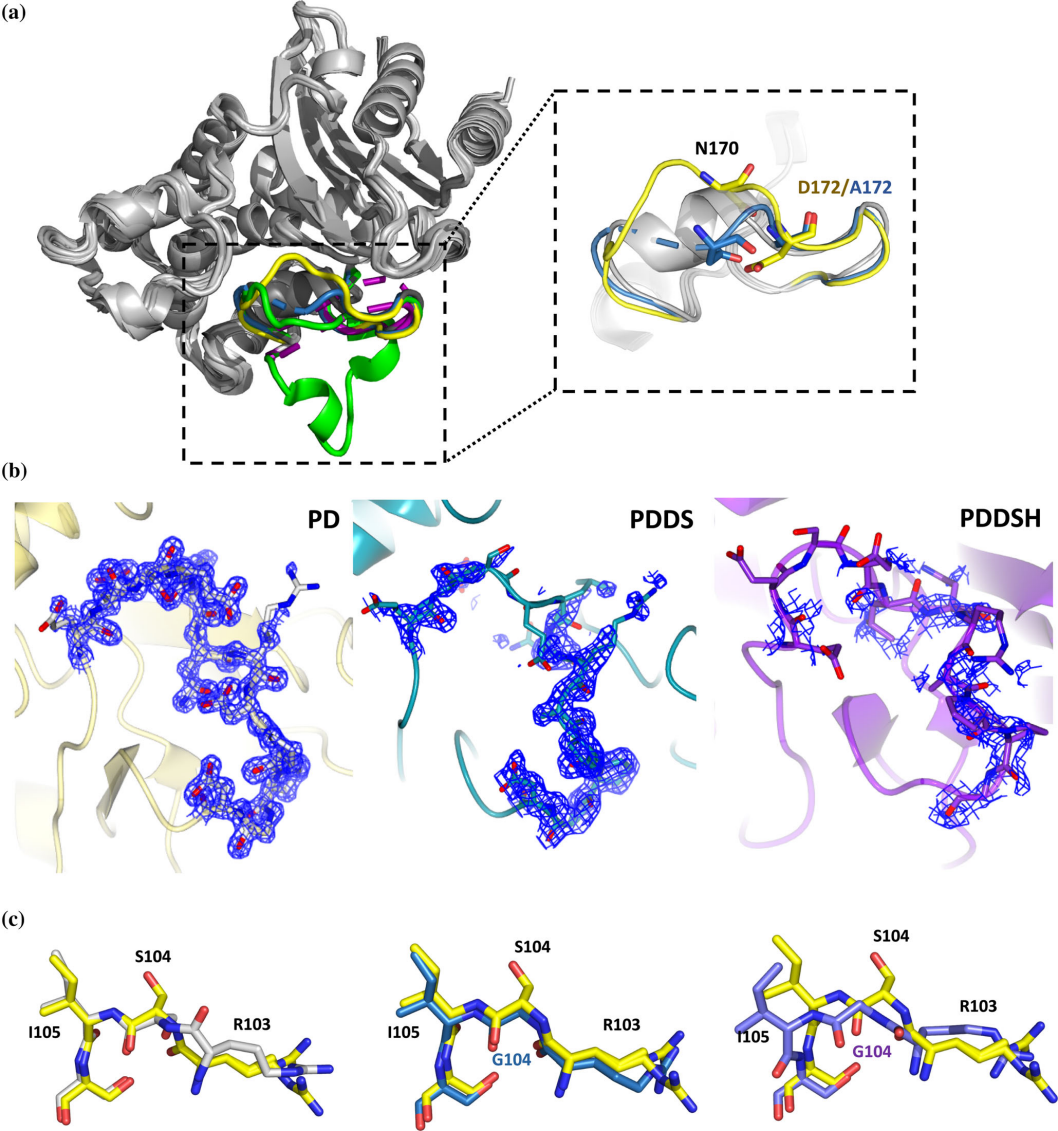
Crystal structures of BlaC P167S/D240G (PD, PDB ID: 9HIT), P167S/D240G/D172A/S104G (PDDS, PDB ID: 9HCK), and P167S/D240G/D172A/S104G/H184R (PDDSH, PDB ID: 9HJ2). (a) An overlay is shown of the crystal structures of Ω‐loops of P167S (green, PDB 8OEI [Sun et al., 2023], two conformations), PD (yellow), PDDS (blue), PDDSH (purple, two conformations) and WT (gray, PDB 2GDN; Wang et al., 2006). Missing density for the backbone is indicated with dashed coils. (b) Electron density of Ω‐loops of PD, PDDS, and PDDSH structures. The well‐ordered Ω‐loop of PD (left) (residues 165 to 175) is indicated by 2|Fo‐Fc| maps (blue), contoured at 1.0 σ. The structures of mutant PDDS (middle) and PDDSH (right) show weak or absent density for the Ω‐loop. The electron density poor region of Ω‐loop in the PDDSH was modeled using the Ω‐loop of the WT. (c) The positions of Arg103, Ser104/Gly104, Ile105, and Ser106 in the gatekeeper loop are shown. The overlay of PD (yellow) and WT (gray) is shown in the left panel. The middle and right panels show the overlays of PD (yellow) with PDDS (blue) and PDDSH (purple), respectively.

The ceftazidime fitness and protein stability increases that are observed during the successive steps of selection indicate a straightforward uphill walk in the evolutionary landscape toward a new fitness peak. To determine the accompanying conformational changes, the mutants were characterized by crystallography and NMR spectroscopy. The Ω‐loop of BlaC P167S was reported to be in two conformations, a semi‐closed and an open state (Sun et al., 2023). The crystal structures of BlaC variants PD, PDDS, and PDDSH were solved at 1.3, 1.6, and 1.4 Å resolution, respectively. While the refinement statistics for the PD and PDDS structures are within the expected values for the resolution (R_free_ of 18 and 17, respectively, Table S3), the PDDSH dataset suffers from translational non‐crystallographic symmetry, resulting in a higher R_free_ value of 25. Between these mutants, significant differences are observed in the Ω‐loop (Figure 3). For BlaC PD, the Ω‐loop exhibits a large conformational change compared to WT, resulting in a significant expansion of the active site cavity (Figure 3a, yellow), in line with the changes observed for BlaC P167S (green), which were attributed to the *cis*‐to‐*trans* change in the peptide bond between residues 166 and 167 (Sun et al., 2023). In PDDS (blue), the loss of the negatively charged carboxyl group of Asp172 (to Ala) causes disruption of the low‐barrier H‐bond of D179‐D172, which was shown before to stabilize the Ω‐loop in WT BlaC (Sun et al., 2024). As a consequence, the chain between residues 167 and 172 is only partially ordered, indicating mobility in this region of the Ω‐loop (Figure 3b). In the BlaC variant PDDSH (purple in Figure 3a), the Ω‐loop residues 165–172 are disordered, indicating still more conformational flexibility (Figure 3b). In this variant, the peptide bond between Gly104 and Ile105 is flipped, and the gatekeeper loop moves 2.5 Å compared to the PD and PDDS variants, enlarging the active site (Figure 3c). Thus, during this evolutionary trajectory, disorder in the Ω‐loop increases, whereas the rest of the structure remained similar.

**FIGURE 4:**
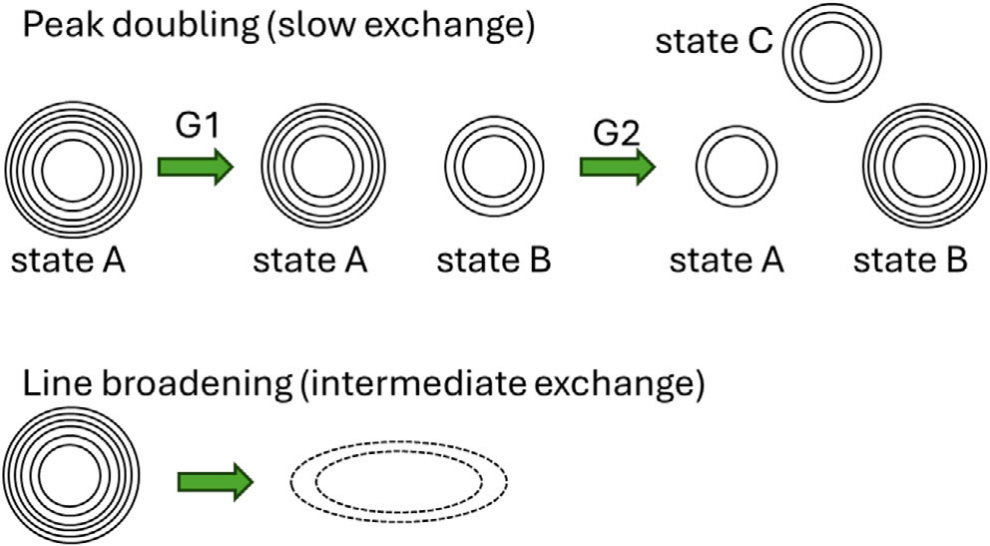
Schematic illustration of the effects on the NMR peaks (shown as contours) due to the presence of multiple conformations. For conformations in slow exchange, peak doubling is observed; for intermediate exchange, large line broadening occurs, and signals become undetectable.

**FIGURE 5:**
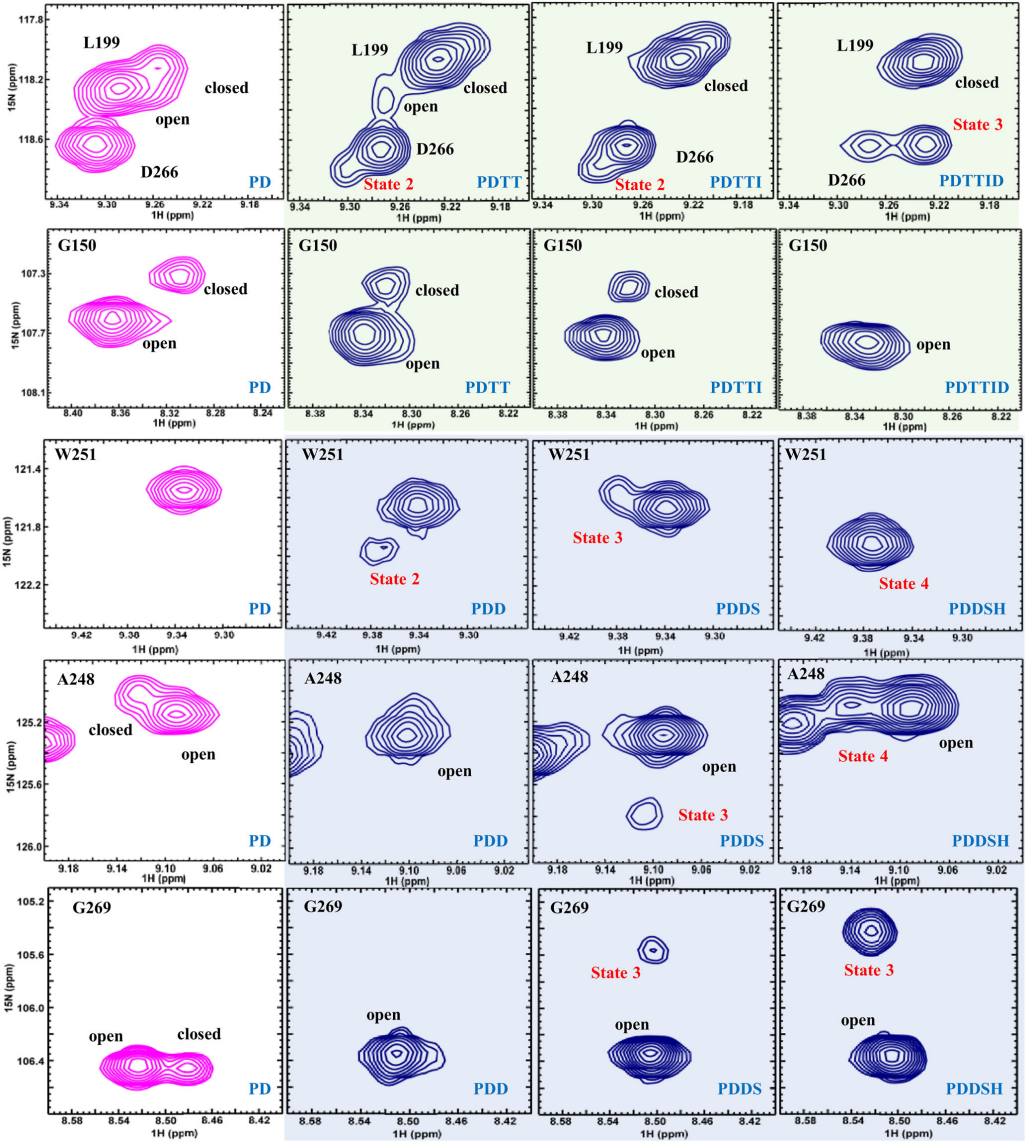
Details of ^1^H‐^15^N TROSY‐HSQC spectra showing resonances for residues of BlaC P167S/D240G (magenta) and evolved BlaC mutants (navy). The green and blue backgrounds represent the variants that were selected at 23°C and 37°C, respectively. The open and closed states refer to those described for BlaC P167S (Sun et al., 2023).

**FIGURE 6:**
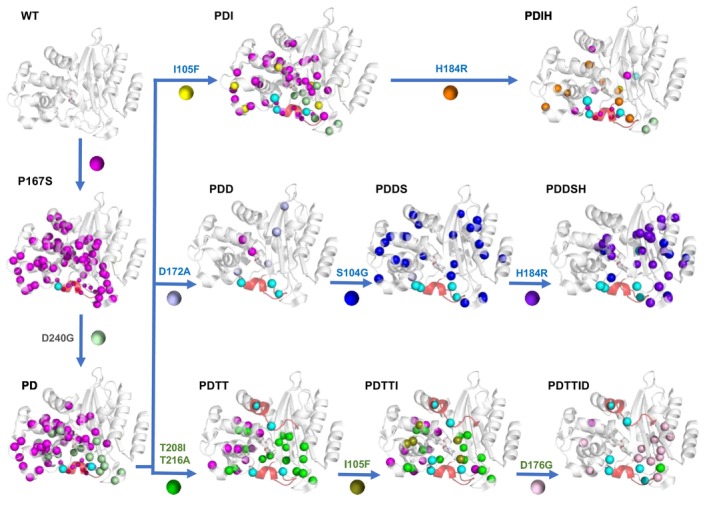
Conformational variations in BlaC upon introduction of mutations based on the NMR results. The nitrogen atoms of amide groups that display peak doubling are shown as spheres. Small spheres in the Ω‐loop indicate that the amides exist in two states, one of which is broadened beyond detection (as inferred from halved intensities) (Sun et al., 2023). Residues with missing amide signals are indicated in red cartoon. In WT BlaC, no peak doubling is observed. The mutant P167S was reported before to show amides with double peaks, indicated in magenta (Sun et al., 2023). In BlaC PD part of the peak doubling pattern is the same as in P167S and part has changed (pale green). Introduction of additional mutations found in the directed evolution branches changes the peak doubling and broadening patterns in each case. The mutation sites are shown in cyan.

Crystallization tends to select a single conformation from multiple states in solution, and only the absence of electron density (disorder) is an indication of conformational diversity. NMR spectroscopy is sensitive to conformational exchange, and the effects on the spectrum depend on the rate of exchange (Figure 4). The ^1^H–^15^N TROSY‐HSQC spectra were assigned for all mutants, using HNCA experiments and the available assignments for WT BlaC (Elings et al., 2017). Chemical shift perturbations were observed in large parts of the protein as a consequence of the mutations (Figure S7). The signals of the amides of the Ω‐loop are missing, indicating that they sample different conformations that exchange on the intermediate‐fast timescale (100 s^−1^). Furthermore, peak doubling occurs in many of the mutants. The appearance of two peaks for one amide group indicates that it occurs in two states that exchange slowly (<<50 s^−1^). We analyzed the doubling effects by comparing, for each successive mutant with its predecessor, whether for an amide resonance doubling remained present and whether the relative orientation of the peaks remained the same. If the resonance of an amide is still doubled but at least one of the two peaks is in a different position, the amide experiences a new state (see examples in Figure 5). In Figure 6, the residues with missing signals are indicated in red cartoon representation, and amide nitrogen atoms that show peak doubling are plotted as spheres on the structure of WT BlaC. State changes for each nitrogen atom are indicated in different colors. A remarkably complex behavior of peak doubling and intermediate exchange is observed. It is clear that in most mutants a large number of amides experience more than a single state. Furthermore, new pairs of conformations are accessed in each mutant. In some cases, a mutation leads to peak doubling in large regions, suggesting conformational changes that involve a large part of the protein (for example, P167S and S104G), whereas others cause new local conformations to be populated (D240G and I105F). Some mutations stabilize a single conformation, largely eliminating the peak doubling (D172A), and others stabilize one part of the protein and introduce new peak doubling and, consequently, new conformational states, in other regions (H184R). The effect of a mutation also depends on its background (epistasis), as is clear for mutation H184R, which occurred in two branches of the directed evolution trajectory.

## 3 DISCUSSION

Selection for gain‐of‐function of an enzyme via directed evolution is often accompanied by destabilization (Bloom & Arnold, 2009; Fasan et al., 2007; Fasan et al., 2008; Socha & Tokuriki, 2013; Stimple et al., 2020; Tokuriki et al., 2008; Tokuriki & Tawfik, 2009). Stability loss is also clearly observed when selecting for enhanced ceftazidime activity in BlaC, as indicated by a decrease in the melting temperature of more than 9°C for the PD mutant and more than 11°C for the PDTT mutant. In the next generations, stability was regained while, simultaneously, activity was further enhanced. The *T*_m_ values were measured for purified protein in vitro, reflecting intrinsic stability under defined conditions and cannot be translated directly to stability in the complex environment of the bacterial periplasm. However, we have noted before a clear correlation between an increase in β‐lactamase *T*_m_ and protein level in the periplasm (Radojković et al., 2025).

Directed evolution was performed by selecting for increased ceftazidime resistance at both 23°C and 37°C, to investigate how temperature shapes adaptive pathways, stability, and function in BlaC. MIC determination was conducted at the corresponding selection temperatures to enable correlation between the selection environment and resistance phenotype. Variants selected at 37°C display increased antibiotic resistance but did not regain WT‐like stability, suggesting that enhanced activity can be achieved despite stabilization loss. In contrast, variants selected at 23°C initially underwent significant stability loss but partially regained thermal stability through compensatory mutations over successive generations. These findings align with previous reports indicating that elevated temperatures intensify selection against protein instability and restrict mutational space, whereas lower temperatures permit exploration of alternative or more destabilizing adaptive pathways (Berezovsky & Shakhnovich, 2005; Tokuriki et al., 2007; Tokuriki & Tawfik, 2009). Kinetic analyses were performed primarily at 25°C for comparability with the literature, with additional measurements in the range from 10°C to 35°C to assess the temperature dependence of catalysis. The temperature dependence profiles of the mutants selected at 23°C or 37°C (Figure 2) do not show clear differences.

It is well‐established that enhanced flexibility of the Ω‐loop of β‐lactamases can lead to higher ceftazidime activity, because opening of the active site facilitates access for this large substrate (Brown et al., 2020; Sun et al., 2023; Yi et al., 2016). Conformational changes in this loop also lead to displacement of the catalytic residue Glu166, which appears not to be required for ceftazidime hydrolysis (He et al., 2020; Sun et al., 2024; Tooke et al., 2023), contrary to what is the case for penicillins and first and second generation cephalosporins (Pemberton et al., 2020; Strynadka et al., 1992). The initial mutations found in the selection screens are located in or close to the Ω‐loop (P167S, D240G), and also in later rounds such mutations were found (D172A, D176G). Some have been described before to destabilize the loop (Ghiglione et al., 2018; Novais et al., 2010; Papp‐Wallace et al., 2017; Patel et al., 2015; Poirel et al., 2001; Stojanoski et al., 2015). Also, two mutations in the gatekeeper loop were found (S104G and I105F). As the name suggests, this loop covers part of the entry to the active site and lies over the Ω‐loop (Feiler et al., 2013), and these mutations further enhance the accessibility of the active site (Poirel et al., 2001; Radojković & Ubbink, 2024; Yi et al., 2012). Three mutations are found far away from the Ω‐loop (H184R, T208I, and T216A), two of which are also far from the active site. His184 and Thr208 both have a Cα‐Cα distance to the catalytic Ser70 between 16 and 17 Å. The intermediate exchange regime observed in the NMR spectra shows that the double mutation T208I/T216A leads to flexibility of the α‐helix and connecting loop in which these two residues are located, which suggests that it opens up the active site on the opposite side of the Ω‐loop. Mutation H184R occurred twice, in different branches of the directed evolution experiments and in both cases causes an increase in the melting temperature, suggesting, therefore, that this mutation is generally stabilizing. In another laboratory evolution experiment, our group identified the same mutation in a search for global stabilizing mutations (Radojković et al., 2025). An Arg at position 184 could potentially form salt bridges with Glu59 and Asp63 and a cation‐π interaction with Tyr48.

**FIGURE 7:**
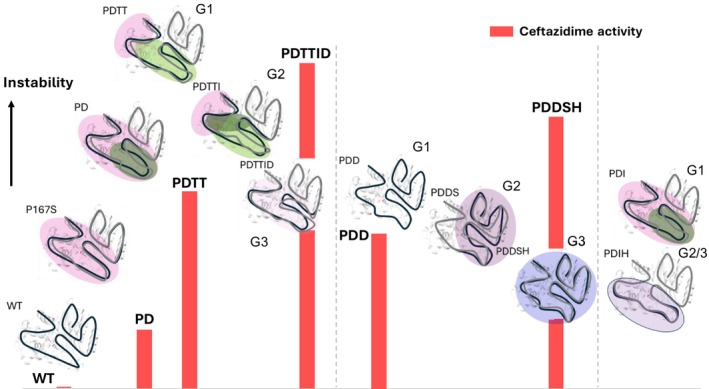
Schematic representation of conformational states. For each BlaC variant conformational states are indicated as black and gray curves. In protein regions in which peak doubling is observed, the black and gray curves differ, highlighted by the colored ovals. The light pink, purple, and blue ovals schematically indicate the presence of distinct states, as evidenced by regions exhibiting coherent peak doubling of amides. The higher the position in the graph, the lower the melting point of the variant, indicating its instability. Note the variety of conformational states among the variants. The red bars represent the relative catalytic turnover number (Table S2) for ceftazidime hydrolysis. The vertical dashed lines separate the different branches of the directed evolution tree.

For one branch of the directed evolution experiment, we managed to obtain crystal structures of the successive mutants, PD, PDDS, and PDDSH, showing the opening and increasing disorder of the Ω‐loop in an otherwise defined protein structure, suggesting minor structural changes. Campbell et al. reported a similar series of crystal structures of enzymes along a directed evolution pathway and also found minimal changes in atomic positions, but significant differences in atomic interactions and H‐bond networks. Furthermore, they observed increased dynamics in certain loops and loss of unproductive motions (Campbell et al., 2016). The NMR spectra paint a complex picture, showing dynamics on μs–ms timescales in several regions around the active site (red cartoons in Figure 6), as well as a remarkable variety of peak doubling patterns, indicating that the directed evolution strongly affected the internal dynamics of BlaC. Observation of dynamics on several timescales of evolved enzymes was also reported by Otten et al. for a system in which dynamics were essential for catalysis (Otten et al., 2018), and González et al. showed how μs–ms dynamics can be optimized in directed evolution (González et al., 2016). In the P167S and PD variants, peak doubling is observed for many amides, indicating that a large part of the protein populates two states. Introduction of every next mutation changes the distribution of states, introduces new states in parts of the protein and, in some cases, leads to a single state for most of the amides. This diversity of states seems uncorrelated with the stability. To illustrate the results, a schematic is shown in Figure 7 in which black and gray lines schematically represent conformational states in various regions of the protein with coherent peak doubling behavior (indicated with colored ovals). The vertical position of the mutant represents the instability, using the melting temperature as a proxy, and the red bars indicate the relative turnover number of ceftazidime (Table S2). WT BlaC is stable and in a single, well‐defined conformation. Upon disruption of the Ω‐loop, the protein enters a region in the conformational space in which many conformations are easily accessible and single mutations readily lead to the population of new states. The most active variants (G3) have not regained a single, well‐defined state.

The NMR spectra are sensitive to the presence of different states but give no information about what they look like. Peak doubling mostly results in resonances close together in the spectrum, suggesting that the conformational changes are not large, though the slow exchange regime indicates that the energy barriers between them are sizable. The conformational changes may be too small to be detected by x‐ray crystallography, or the crystallization process may favor one of them. Even if the conformational states differ only slightly, the large variation is striking, given that WT BlaC is in a single conformational state. Our results indicate that additional states are readily accessed upon the introduction of a few mutations, yet in NMR spectra of native, folded proteins, extensive peak doubling is exceptional. That observation suggests that the natural selection pressure for a single, well‐defined state must be strong; otherwise, multiple states would be observed also for native proteins.

## 4 CONCLUSIONS

Directed evolution is an effective method to enhance specific functions of enzymes. Selection is based on a single parameter; an increase in activity and a gradual gain in successive generations is usually observed. This process is often described as a climb to the top of a fitness landscape. As native enzymes generally have a well‐defined structure with a single conformation, naively, it can be assumed that each enzyme variant along the evolutionary trajectory is also in a single conformation; in other words, that the conformation landscape resembles the fitness landscape. Alternatively, binary models are used, in which a gradual population change occurs from a defined inactive conformation to an active one. This picture is too simple for the case reported here. The conformational landscape is much more complex, with multiple global or local conformational states being populated in the successive steps, without a clear relationship between stability and restriction of the conformational space. The optimized enzymes have not (yet) attained a single conformation. It will be of interest to determine whether such variety of conformations is observed also in the directed evolution of other enzymes as well as in natural evolution, for example, by using resurrected ancestral proteins.

## 5 MATERIALS AND METHODS

### 5.1 Construction of a random mutant library

The initial BlaC mutant library was constructed by carrying out error‐prone polymerase chain reactions (EP‐PCR) using the *blaC* gene as the template (Sun et al., 2023). The gene encodes an *E. coli* expression‐optimized *blaC* gene with a TAT signal sequence for translocation to the periplasm. Only the gene coding for the mature enzyme, not the signal sequence or the vector, was amplified and mutated. For the directed evolution, plasmids from clones selected from a preceding evolutionary round were subjected to EP‐PCR with a GeneMorph II Random Mutagenesis Kit. Initial denaturation was performed at 95°C for 2 min, followed by 30 cycles of denaturation at 95°C for 30 s, annealing at 60°C for 30 s, and extension at 72°C for 3 min. The DNA concentration of the libraries was normalized by estimating the PCR yield based on gel band intensity in comparison with that of the 1.1‐kb standard.

### 5.2 Selection on plates

The mutated *blaC* gene libraries were cloned behind the *lac* promoter in pUK21 through restriction and ligation using *Bgl*II and *Xho*I restriction sites. *E. coli* KA797 cells were transformed with the plasmid library and plated on lysogeny broth (LB) agar plates with kanamycin (50 μg/mL), isopropyl β‐d‐1‐thiogalactopyranoside (IPTG, 1 mM), and varying concentrations of ceftazidime (1, 10, 25, 40, and 60 μg/mL). The plates for the template were incubated at 23, 30, or 37°C for 3 days, 18 h, or 12 h, respectively. Approximately 5 × 10^5^ clones were screened at each temperature, and three *blaC* clones were found on the plate incubated at 30°C. For the following directed evolution selection, plates were incubated at 23 or 37°C. Approximately 1 × 10^7^ clones were screened at each temperature per selection round. Colonies were transferred to LB liquid cultures and incubated overnight at 37°C and 250 rpm. Plasmids were isolated and transformed into fresh *E. coli* KA797 cells to confirm that resistance was the result of mutations in the *blaC* gene and not due to changes in the host cell. The mutations were identified by DNA sequencing.

### 5.3 Antimicrobial susceptibility testing

Antibiotic resistance was tested in the *E. coli* cells carrying pUK21‐*blaC* plasmids with the gene behind the *lac* promoter. Ten‐microliter drops of *E. coli* cultures with optical densities (OD_600_) of 0.3, 0.03, 0.003, and 0.0003 were applied on the LB plates containing 50 μg/mL kanamycin (to maintain the plasmid), 1 mM IPTG, and varying concentrations of ceftazidime (0.5–65 μg/mL). The MIC was determined using a drop assay. The plates were incubated at 23 or 37°C until growth was visible and imaged using a Gel Doc XR+ (Bio‐Rad) and ImageLab version 6.0.1 (Bio‐Rad).

### 5.4 BlaC production

The genes for all BlaC mutants were cloned into pET28a + plasmids downstream of an N‐terminal His‐tag with T7 promoter and a TEV cleavage site by using the Gibson assembly method. The proteins were produced and purified according to previously described protocols (Elings et al., 2017). Pure protein was stored at −80°C in 100 mM sodium phosphate (pH 6.5). For determination of the amount of soluble protein, the OD_600_ of the overnight culture was adjusted to 1, and 500 μL was centrifuged and resuspended in 50 μL B‐PER (Thermo Scientific) for lysis. After 30 min of incubation at room temperature, 30 μL was treated with SDS‐PAGE cracking buffer (20 mM Tris/HCl pH 6.8, 5 mM EDTA, 0.5% SDS, 0.1% β‐mercaptoethanol). The samples were centrifuged for 3 min. The soluble and lysate fraction samples were analyzed by running on a 4%–15% Mini‐PROTEIN TGX Stain Free Protein SDS‐PA gel. The signal intensity of the band corresponding to BlaC for each mutant was compared to the signal intensity of WT BlaC using ImageLab software (BioRad). The experiment was performed in triplicate.

### 5.5 Thermal stability

The thermal stability of BlaC mutants was determined by tryptophan fluorescence changes as a function of temperature using a Tycho NT.6 (NanoTemper Technologies, München, Germany). The fluorescence ratio at 330 and 350 nm was used to determine the melting temperature. All measurements were done in triplicate in 100 mM sodium phosphate buffer at pH 6.5.

### 5.6 Enzyme kinetics

Kinetic parameters on BlaC mutants were determined by monitoring the absorption change at 486 nm for nitrocefin hydrolysis (Δε_486_ = 18/mM/cm) (van Alen et al., 2023) with a TECAN infinite® M1000PRO plate reader at 25°C for 5 min. The initial velocities were fitted to the Michaelis–Menten equation:

(1) $$v_{i}=\frac{k_{\mathrm{cat}}\left[E\right]\left[S\right]}{K_{M}+\left[S\right]}$$

where $v_{i}$ is the initial velocity, *k*_cat_ is the turnover number, *K*_M_ is the apparent Michaelis constant, [*S*] is the initial concentration of nitrocefin (0, 10, 20, 50, 100, 200, 300, and 400 μM) and [*E*] the concentration of BlaC (200 nM). The nitrocefin hydrolysis by WT BlaC (2 nM) was measured on a PerkinElmer Lambda 1050+ UV–Vis spectrometer at 25°C. The hydrolysis reaction comprises several steps, so the definition of *K*_M_ differs from the standard one and is thus considered an apparent *K*_M_. To study ceftazidime hydrolysis, absorbance change at 260 nm (Δε_260_ = 7 ± 1 mM^−1^ cm^−1^) was measured for 5 min using 1 μM of BlaC and 35 μM ceftazidime at 10, 15, 20, 25, 30, or 35°C on a temperature‐controlled PerkinElmer Lambda 1050+ UV–Vis spectrometer. All kinetic measurements were performed in duplicate in 100 mM NaPi buffer (pH 6.5).

### 5.7 NMR spectroscopy

Standard HNCA and TROSY‐HSQC experiments were recorded using [^15^N, ^13^C] labeled BlaC mutants on a Bruker AVIII HD 850 MHz spectrometer with a TCI cryoprobe at 25°C in 100 mM sodium phosphate buffer (pH 6.5), with 1 mM TSP (trimethylsilylpropanoic acid) and 6% D_2_O in a Shigemi tube. Data were processed with Topspin 4.1.1 (Bruker Biospin) and analyzed using CcpNmr version 2 (Vranken et al., 2005). Assignments were based on those for the WT spectra at pH 6.5 (BMRB ID: 27067) (Elings et al., 2017), BlaC P167S spectra at pH 6.5 (BMRB ID: 51876) (Sun et al., 2023), and confirmed with HNCA spectra. The assignments for BlaC PD, PDD, PDDS, PDDSH, PDI, PDIH, PDTT, PDTTI, and PDTTID have been deposited in the BMRB under access codes 52517, 52518, 52679, 52680, 52631, 52686, 52683, 52684, and 52685, respectively. The average chemical shift differences ∆δ between the two states observed for BlaC of the ^1^H ($∆\delta_{1}$) and ^15^N $\left(∆\delta_{2}\right)$ resonances of backbone amides were calculated with Equation (2).

(2) $$∆\delta =\sqrt{\frac{1}{2}\left[∆{\delta_{1}}^{2}+{\left(\frac{∆\delta_{2}}{5}\right)}^{2}\right]}$$

### 5.8 Protein crystallization

Crystallization conditions for BlaC PD, PDDS, and PDDSH (12 mg mL^−1^) were screened by the sitting‐drop method using the JCSG^+^, BSC, PACT, and Morpheus screens (Molecular Dimensions, Catcliffe, UK) at 20°C with 200 nL drops with a 1:1 protein to screening condition ratio. Crystals of PD grew in 0.1 M sodium cacodylate at pH 6.07 with 1.16 M trisodium citrate as precipitant. A crystal of PDDS grew within 2 weeks in 0.1 M Bis‐Tris buffer, pH 5.5, and 2 M (NH_4_)_2_SO_4_ as additive. Crystals of PDDSH grew in 0.1 M MES buffer, pH 6.3, and with 0.864 M trisodium citrate as additive. After 1 month, the crystals were mounted on cryoloops in mother liquor, with the addition of 30% glycerol and flash frozen in liquid nitrogen for x‐ray data collection.

### 5.9 Data collection, processing, and structure refinement

Diffraction data for PD were collected at the Diamond Light Source (DLS, Oxford, England, Project MX25413). The PDDS and PDDSH structures were collected at the European Synchrotron Radiation Facility on the MASSIF beamline (Project MX2526). The resolution cutoff was determined based on completeness and CC1/2 values. The data were processed by XDS (Kabsch, 2010) and scaled using Aimless (Evans, 2011). The structures were solved by molecular replacement using MOLREP (Winn et al., 2011) from the CCP4 suite (Winn et al., 2011) with PDB entry 2GDN (Wang et al., 2006) as model for molecular replacement. Model building and refinement were performed in Coot and REFMAC (Winn et al., 2011). Waters were added in REFMAC during refinement. The models were further optimized using the PDB‐REDO web server (Joosten et al., 2014). The refinement and data collection statistics are given in Table S3. The structures for BlaC PD, PDDS, and PDDSH have been deposited in the Protein Data Bank (PDB ID: 9HIT, 9HCK, and 9HJ2).

## AUTHOR CONTRIBUTIONS

**Jing Sun:** Conceptualization; methodology; data curation; investigation; validation; formal analysis; funding acquisition; visualization; writing – original draft; writing – review and editing. **Monika Timmer:** Methodology; investigation. **Steffen Brünle:** Formal analysis; validation; writing – review and editing. **Aimee L. Boyle:** Writing – review and editing. **Marcellus Ubbink:** Conceptualization; formal analysis; supervision; resources; project administration; visualization; funding acquisition; writing – original draft; writing – review and editing; methodology.

## Supporting information


**Data S1.** Supporting Information.

## Data Availability

The data that support the findings of this study are available from the corresponding author upon reasonable request.
