# Supplementary material for: Directed evolution of a beta‐lactamase samples a wide variety of conformational states
Source: Protein Sci. 2025 Oct 11;34(11):e70322. doi: 10.1002/pro.70322 (PMC12514840; doi:10.1002/pro.70322)
Supplement: Supplementary file 1 — Data S1. Supporting Information. [file PRO-34-e70322-s001.docx]

**Supplementary Information of**

**Directed evolution of the beta-lactamase BlaC samples a variety of conformational states**

Jing Sun, ^a^ Monika Timmer, ^a^ Steffen Brünle,^b^, Aimee L. Boyle, ^a, c^ Marcellus Ubbink ^a, *^

**^a^**Macromolecular Biochemistry, Leiden Institute of Chemistry, Leiden University, Einsteinweg 55, 2333 CC Leiden, The Netherlands.

^b^Biophysical Structure Chemistry, Leiden Institute of Chemistry, Leiden University, Einsteinweg 55, 2333 CC Leiden, The Netherlands.

^c^ Current address: School of Chemistry, University of Bristol, Cantock's Close, Clifton, Bristol BS8 1TS, United Kingdom

**^*^**Corresponding author at Macromolecular Biochemistry, Leiden Institute of Chemistry, Leiden University, Einsteinweg 55, 2333 CC Leiden, The Netherlands. Email address: [m.ubbink@chem.leidenuniv.nl](mailto:m.ubbink@chem.leidenuniv.nl) (M. Ubbink)

**Supplementary Figures**

**Fig S1.** Drops of increasing dilution of *E. coli* cultures were spotted on LB-agar plates containing ceftazidime and kanamycin (50 µg mL^-1^) to ensure plasmid stability and 1 mM IPTG to induce gene expression. The plates were incubated at 37 ℃ and 23 ℃ until growth was visible. The letters refer to the variants (Table 1). The light green background represents the variants that evolved at 23 ℃ and the light blue and orange refer to the variants that evolved at 37 ℃. The red square is the template used for direct evolution.

**Fig S2.** Activity against ceftazidime of evolved BlaC mutants at 37 ℃, wild-type BlaC, and inactive S70A BlaC as the negative control. Cultures of *E. coli* expressing genes of the BlaC variants were spotted at increasing dilution on the plates containing various concentration of ceftazidime. All the plates contain kanamycin (50 µg mL^-1^) to ensure plasmids stability and 1 mM IPTG to induce gene expression.

**Fig S3.** Activity against ceftazidime of evolved BlaC mutants at 23 ℃, wild-type BlaC, and inactive S70A BlaC as the negative control. Cultures of *E. coli* expressing genes of the BlaC variants were spotted at increasing dilution on the plates containing various concentrations of ceftazidime. All the plates contain kanamycin (50 µg mL^-1^) to ensure plasmids stability and 1 mM IPTG to induce gene expression.

**Fig S4.** Protein production and melting temperature **a)** The histogram and SDS-PAGE gel displays the levels of soluble BlaC variants relative to wild-type BlaC in the cytoplasm. The black arrow shows the position of BlaC at 31 kDa, the red arrow shows the position of the *E. coli* protein that was used for calibration of the density in each lane. **b)** The derivative signal of the Trp fluorescence ratio at 350 and 330 nm as a function of temperature for the BlaC variants. The melting temperatures are indicated.

**Fig S5.** Chemical structures of nitrocefin and ceftazidime.

**Fig S6.** Kinetics. **a)** Activity curves for nitrocefin hydrolysis at 25 °C in NaPi buffer pH 6.5 (Table S1); **b)** The progress curves of ceftazidime hydrolysis at an absorbance at 260 nm for BlaC PDTTID (left) and PDDSH (right) at increasing temperature.

**Fig S7.** CSPs relative to the chemical shifts of the amides in BlaC PD (a) or WT (b) are plotted on the structure of PD variant (PDB:9HIT) or WT BlaC (PDB:2GDN)(Wang et al., 2006), respectively for the indicated variants. Backbone amides are indicated as spheres. The cyan spheres represent the mutation sites. Blue: CSP ≤ 0.05 ppm, Yellow: 0.05 < CSP ≤ 0.1 ppm, orange: 0.1 < CSP ≤ 0.15 ppm, red: CSP > 0.15 ppm, grey: peaks that cannot be detected or unassigned, black: no data available (proline).

**Table S1.** Kinetic parameters for nitrocefin hydrolysis by BlaC variants at 25 °C. The errors are the standard deviation of the mean over three experiments. The reduction is for the *k*_cat_/*K*_M_^app^ values of the mutants relative to WT BlaC.

| BlaC variants | *k*_cat_  (s^-1^) | $K_{M}^{\mathrm{app}}$  (µM) | *k*_cat_/$K_{M}^{\mathrm{app}}$  (10^5^ M^-1^s^-1^) | Reduction  cp. to WT |
| --- | --- | --- | --- | --- |
| Wildtype (WT) | 120 ± 8 | 215 ± 19 | 5.6 ± 0.6 |  |
| P167S/D240G | 5.2 ± 0.8 | 43 ± 6 | 1.2 ± 0.3 | 4.7 ± 1.3 |
| P167S/D240G/T208I/T216A | 0.4 ± 0.1 | 68 ± 3 | 0.07 ± 0.02 | 80 ± 24 |
| P167S/D240G/T208I/T216A/I105F | 0.40 ± 0.08 | 53 ± 1 | 0.08 ± 0.02 | 70 ± 19 |
| P167S/D240G/T208I/T216A/I105F/D176G | 0.15 ± 0.01 | 41 ± 3 | 0.036 ± 0.001 | 155 ± 17 |
| P167S/D240G/D172A | 0.38 ± 0.03 | 41 ± 11 | 0.10 ± 0.02 | 56 ± 13 |
| P167S/D240G/D172A/S104G | 0.11 ± 0.01 | 14 ± 6 | 0.08 ± 0.02 | 70 ± 19 |
| P167S/D240G/D172A/S104G/H184R | 0.23 ± 0.03 | 39 ± 10 | 0.06 ± 0.01 | 93 ± 18 |
| P167S/D240G/I105F/H184R | 7.87 ± 0.04 | 519 ± 5 | 0.15 ± 0.01 | 37 ± 5 |

**Table S2.** Catalytic turnover numbers as defined in the main text for ceftazidime hydrolysis by wild-type BlaC and several variants at different temperatures. The errors are the standard deviation of the average over duplicate experiments.

| BlaC variants | 10℃ | 15℃ | | 20℃ | | | 25℃ | | 30℃ | | 35℃ | |
| --- | --- | --- | --- | --- | --- | --- | --- | --- | --- | --- | --- | --- |
|  | | | | | | $\frac{[P]}{time\left( s \right)\cdot\left[ E \right]}$ (s^-1^) | | | | | | |
| Wildtype (WT) |  | |  | |  | | | 0.002 ± 0.001 | |  | |  |
| P167S/D240G | 0.011 ± 0.006 | 0.06 ± 0.01 | | 0.09 ± 0.01 | | | 0.11 ± 0.01 | | 0.14 ± 0.02 | | 0.22 ± 0.01 | |
| P167S/D240G/T208I/T216A | 0.17 ± 0.02 | 0.21 ± 0.01 | | 0.32 ± 0.01 | | | 0.39 ± 0.01 | | 0.48 ± 0.05 | | 0.62 ± 0.02 | |
| P167S/D240G/T208I/T216A/I105F/D176G | 0.35 ± 0.01 | 0.36 ± 0.03 | | 0.51 ± 0.05 | | | 0.61 ± 0.02 | | 0.78 ± 0.03 | | 0.83 ± 0.06 | |
| P167S/D240G/D172A | 0.12 ± 0.02 | 0.16 ± 0.01 | | 0.21 ± 0.01 | | | 0.30 ± 0.01 | | 0.35 ± 0.01 | | 0.40 ± 0.02 | |
| P167S/D240G/D172A/S104G/H184R | 0.25 ± 0.01 | 0.38 ± 0.03 | | 0.43 ± 0.04 | | | 0.51 ± 0.02 | | 0.66 ± 0.03 | | 0.67 ± 0.04 | |

**Table S3.** Data collection and refinement statistics for the structures of BlaC mutants.

| Data collection | P167S/D240G | | P167S/D240G/D172A/S104G | P167S/D240G/D172A/S104G/H184R |
| --- | --- | --- | --- | --- |
| Wavelength (Å) | 0.98Å | | 0.87Å | 0.97 Å |
| Resolution (Å) | 38.87 (1.32) 1.30-1.32 | | 38.44 (1.6) 1.60-1.63 | 53.55 (1.43) 1.40-1.43 |
| Space group | P1 21 1 | | P1 21 1 | P1 21 1 |
| Unit cell a, b, c (Å) | 38.90 54.45 53.94 | | 38.88 54.64 54.16 | 53.81 57.27 76.92 |
| Unit angle α, ß, γ | 90.0 92.4 90.0 | | 90.0 93.1 90.0 | 90.0 95.7 90.0 |
| CC1/2 | 99.6 (54.8) | | 99.8 (86.1) | 97.5 (33.5) |
| Rpim (%) | 6 (55.1) | | 3.4 (29.2) | 11.9 (92.2) |
| \|I/σI\| | 7.1 (2.1) | | 14.7 (1.5) | 5.3 (0.3) |
| Completeness (%) | 99.5 (97.7) | | 95.3 (7.8) | 95.1 (65.1) |
| Multiplicity | 3.1 | | 1.8 | 1.9 |
| Unique reflections | 55085 | | 20155 | 87116 |
| Refinement |  |  | |  |
| Atoms protein/ligands/water | 2073/13/323 | 1968/2/291 | | 3817/38/290 |
| B-factors protein/ligands/water (Å^2^) | 12/9/27 | 19/52/26 | | 13/25/21 |
| R_work_/R_free_ (%) | 15.4/17.9 | 14.2/17.3 | | 23.3/25.1 |
| Bond lengths RMSZ/RMSD (Å) | 0.794/0.011 | 0.67/0.01 | | 0.38/0.01 |
| Bond angles RMSZ/RMSD (Å) | 0.84/1.83 | 0.74/1.46 | | 0.58/1.26 |
| Ramachandran plot preferred/outliers | 256/2 | 247/4 | | 484/6 |
| Ramachandran plot Z-score | 0.049 | -0.289 | | -0.383 |
| Clash score | 2.44 | 1.27 | | 2.61 |
| MolProbity score | 1.03 | 0.85 | | 1.05 |

**References**

1. Wang F, Cassidy C, Sacchettini JC (2006) Crystal structure and activity studies of the Mycobacterium tuberculosis β-lactamase reveal its critical role in resistance to β-lactam antibiotics. Antimicrobial agents and chemotherapy 50:2762-2771.
